# Linking alcohol-involved sexual assault to negative emotional outcomes: the relative mediating roles of shame, self-compassion, fear of self-compassion, and self-blame

**DOI:** 10.3389/fpsyg.2024.1370524

**Published:** 2024-08-15

**Authors:** Sherry H. Stewart, Noelle Strickland, Raquel Noguiera-Arjona, Christine Wekerle

**Affiliations:** ^1^Department of Psychiatry, Dalhousie University, Halifax, NS, Canada; ^2^Mood, Anxiety, and Addiction Comorbidity (MAAC) Lab, Department of Psychology and Neuroscience, Dalhousie University, Halifax, NS, Canada; ^3^School of Psychology, University of Sussex, Falmer, United Kingdom; ^4^Offord Centre for Child Studies, Department of Psychology, McMaster University, Hamilton, ON, Canada

**Keywords:** alcohol-involved sexual assault, posttraumatic stress, anxiety, depression, shame, self-blame, self-compassion, fear of self-compassion

## Abstract

**Introduction:**

Alcohol-involved sexual assault (AISA) survivors who were drinking at the time of the assault may be at risk of internalizing victim-blaming myths and stigma. Cognitive-behavioral models posit the link between AISA and negative emotional outcomes may be explained through maladaptive appraisals and coping – i.e., characterological and behavioral self-blame, shame, low self-compassion (i.e., high self-coldness, low self-caring), and fear of self-compassion.

**Methods:**

Using a cross-sectional design and community sample of younger adults (*N* = 409 Canadians, *M* = 28.2 years old, 51.3% women), we examined these mechanisms’ unique effects in mediating the associations between AISA and posttraumatic stress, general anxiety, and depressive symptoms, respectively.

**Results:**

In terms of gender differences, AISA was more common, self-coldness higher, and general anxiety symptoms more frequent in women, and fear of self-compassion was higher in men. Using structural equation modeling that controlled for gender and the overlap between outcomes, shame emerged as the strongest mediator linking AISA with all emotional outcomes. Fear of self-compassion also partially mediated the AISA-posttraumatic stress symptom association, self-coldness partially mediated the AISA-general anxiety symptom association, and characterological self-blame fully mediated the AISA-depressive symptom association.

**Conclusion:**

Avoidance-based processes, ruminative−/worry-based cognitions, and negative self-evaluative cognitions may be distinctly relevant for AISA-related posttraumatic stress, general anxiety, and depressive symptoms, respectively, after accounting for the overarching mediation through shame. These internalized stigma-related mechanisms may be useful to prioritize in treatment to potentially reduce AISA-related negative emotional outcomes, particularly for AISA survivors with posttraumatic stress, general anxiety, and/or depressive symptoms.

## Introduction

Sexual assault (i.e., non-consensual sexual contact and/or violation of sexual integrity) can lead to negative emotional outcomes, including posttraumatic stress, general anxiety, and depressive symptoms (Testa et al., 2004; Carey et al., 2018; Gong et al., 2019). About half of sexual assaults involve survivor and/or perpetrator alcohol use, particularly among younger adults given the tendency for heavier drinking during this life stage (Blayney et al., 2016; O’Callaghan and Ullman, 2021). Further, sexual assault where the survivor was drinking (hereafter referred to as alcohol-involved sexual assault [AISA]) predicts negative emotional outcomes, despite the potential stress-response dampening effects of alcohol and/or the tendency for AISAs to be less physically violent (Gong et al., 2019).

Given the established links of AISA with posttraumatic stress, general anxiety, and depressive symptoms (e.g., Carey et al., 2018; Strickland et al., 2019), exploring potential mechanisms is warranted to identify intervention targets. False victim-blaming myths (e.g., the survivor provoked the sexual assault) and stigma may be particularly salient for those who have experienced AISA (Brown et al., 2018). Indeed, AISA survivors perceived more stigma and are blamed more than non-intoxicated survivors which, if internalized, may predict self-blame and shame (Littleton et al., 2009; Brown et al., 2018). The risk of internalized stigma also suggests low self-compassion and fear of self-compassion may be important additional mediators (Miron et al., 2016; Barlow et al., 2017).

Cognitive-behavioral models may help understand the links of AISA with various negative emotional outcomes. The cognitive-behavioral model of posttraumatic stress disorder posits that trauma survivors’ negative, internal, stable appraisals about the cause (e.g., blaming their own character) and subsequent effects of trauma (e.g., fearing of social judgment), and their maladaptive (e.g., avoidance) coping, may underpin the development of posttraumatic stress symptoms (Ehlers and Clark, 2000; Budden, 2009). From a cognitive perspective, negative, character-based appraisals may also be relevant for depressive symptoms that develop following trauma exposure (Clark and Beck, 2010), and rumination and worry about such appraisals, thought to be a strategy to avoid aversive emotions (i.e., avoidance coping), may be relevant for general anxiety symptoms arising following trauma (Rutter and Brown, 2017). As such, there may be indirect links of AISA with posttraumatic stress, general anxiety, and depressive symptoms, through multiple AISA stigma-related cognitive and behavioral mechanisms including self-blame appraisals, shame (appraisals regarding social condemnation), and maladaptive coping (low self-compassion, fear of self-compassion). Accordingly, the current study explored their relative mediating roles in the associations between AISA and posttraumatic stress, general anxiety, and depressive symptoms.

Shame is a physiologically intense, aversive emotional experience accompanied by judgements of the self as inferior or damaged; shame can be contrasted with guilt, which involves regret about behaviors (Tangney et al., 1996). The evolutionary function of shame may be to maintain harmonious communal relationships by motivating adherence to social norms, the perceived violation of which may inspire shame-related appraisals involving fear of social condemnation (Budden, 2009). The theorized role of cognitive appraisals featuring fear of social judgment suggests shame may be an important mechanism linking AISA to negative emotional outcomes, given the potential for self-perceived violations of social norms if AISA-specific stigma is internalized (Budden, 2009). Supporting this, shame is robustly associated with posttraumatic stress symptoms, particularly following stigmatized traumas, and with general anxiety and depressive symptoms (Tilghman-Osborne et al., 2008; Carey et al., 2018; López-Castro et al., 2019). Similarly, adult survivors of child sexual abuse report high shame, and in turn more negative emotional outcomes (Mac Ginley et al., 2019). Sexual assault is also associated with greater shame than physical assault (Aakvaag et al., 2016). In a cross-sectional study of sexually assaulted women, shame, but not guilt, was associated with posttraumatic stress symptoms through negative self-appraisals (Badour et al., 2020), suggesting shame may be an important mechanism linking AISA to negative emotional outcomes. Indeed, shame has been shown to mediate the association between interpersonal trauma, including sexual assault, and posttraumatic stress symptoms (La Bash and Papa, 2014). This work requires replication and extension to examine AISA effects specifically, emotional outcomes beyond posttraumatic stress symptoms (e.g., general anxiety and depressive symptoms), and shame’s mediational role in the context of other potential mediators (e.g., self-blame, low self-compassion, and fear of self-compassion).

An additional mechanism beyond shame may be low self-compassion (i.e., nonjudgmentally, gently relating to oneself; Neff, 2003). According to Neff (2003), the self-compassion construct consists of three bipolar components: mindfulness vs. overidentification, common humanity vs. isolation, and self-kindness vs. self-judgment. In contrast, Gilbert (2010) operationalizes self-compassion as involving activation of the safeness (high self-caring) and deactivation of the threat/defense (low self-coldness) processing systems. Strickland et al. (2022) recently found empirical support for integrating both operationalizations. They demonstrated a hierarchical structure for self-compassion with two higher-order and six lower-order components: (1) higher-order self-caring (comprised of lower-order mindfulness, common humanity, and self-kindness components) and (2) higher-order self-coldness (comprised of lower-order overidentification, isolation, and self-judgement components) (Strickland et al., 2022). Theoretically, self-compassion may be low among AISA survivors since they may struggle to adopt positive, protective, self-compassionate appraisals. Indeed, sexual assault survivors reported higher self-coldness than non-sexual trauma survivors (Williamson, 2019), and adult survivors of childhood maltreatment reported lower self-compassion than those not maltreated (Miron et al., 2016). Furthermore, the absence of self-compassion theoretically contributes to negative emotional outcomes following a traumatic experience like AISA. Indeed, low self-compassion has been shown to be linked to higher posttraumatic stress symptoms among trauma survivors (Winders et al., 2020), and low self-caring and high self-coldness independently predicted higher anxiety and depressive symptoms among AISA survivors, controlling for gender (Strickland et al., 2019). Therefore, low self-compassion (i.e., low self-caring, high self-coldness) could help explain the link between AISA and negative emotional outcomes. Indeed, low self-compassion was found to mediate the cross-sectional association between childhood abuse and posttraumatic stress symptoms (see review by Wong et al., 2022) even when controlling for the mediating role of negative cognitive appraisals (including self-blame) (Barlow et al., 2017). This work requires replication and extension to examine AISA effects specifically, negative emotional outcomes beyond posttraumatic stress symptoms (e.g., general anxiety and depressive symptoms), and low self-compassion’s mediational role in the context of other potential mediators beyond self-blame (e.g., fear of self-compassion).

Fear of self-compassion involves the tendency to fear, or be reluctant to engage in, self-compassionate attitudes and behaviors. Notably, fear of self-compassion is an independent construct and not simply the absence of self-compassion; for example, one can be compassionate toward the self despite fearing negative consequences (Gilbert et al., 2011). Fear of self-compassion may be another potential mediator in the AISA—negative emotional outcomes link (Gilbert et al., 2011), particularly in the case of posttraumatic stress symptoms (see review by Wong et al., 2022). Supporting this, adult survivors of childhood sexual abuse showed higher fear of self-compassion compared to survivors of childhood physical abuse (Miron et al., 2016), suggesting fear of self-compassion may be especially relevant for highly stigmatized trauma, such as AISA. Similarly, fear of self-compassion, but not self-compassion *per se*, mediated the associations between childhood sexual abuse and depressive and posttraumatic stress symptoms, whereas neither self-compassion nor fear of self-compassion mediated the associations between childhood sexual abuse and depressive and posttraumatic stress symptoms (Miron et al., 2016). Together, although low self-compassion may be associated with AISA, fear of self-compassion may be a stronger mechanism linking AISA to negative emotional outcomes, but this requires specific testing with respect to AISA to extend beyond Miron et al.’s (2016) prior work with childhood sexual abuse.

Aligned with cognitive-behavioral models of trauma, self-blame is higher among AISA survivors (particularly women) compared to survivors of sexual assault not involving alcohol (Ehlers and Clark, 2000; Littleton et al., 2009). However, these studies did not distinguish behavioral self-blame, which targets specific actions (e.g., drinking the day of assault), from characterological self-blame, which targets dispositional, stable, character traits (e.g., being too trusting; Janoff-Bulman, 1979). Given characterological self-blame appraisals are perceived as unchangeable, they may predict more negative emotional outcomes following AISA than behavioral self-blame (Ehlers and Clark, 2000). Accordingly, self-blame failed to significantly mediate the association between AISA and posttraumatic stress symptoms in a two-wave longitudinal study when characterological and behavioral self-blame were undifferentiated (Blayney et al., 2016). However, when differentiated, AISA survivors reported higher characterological self-blame than survivors of sexual assault not involving alcohol, and characterological, but not behavioral, self-blame mediated the link between AISA and higher posttraumatic stress symptoms in a three-wave longitudinal study (Peter-Hagene and Ullman, 2018). Likewise, while behavioral and characterological self-blame, and disengagement coping simultaneously mediated the cross-sectional association between low self-compassion and posttraumatic stress symptoms, only characterological self-blame mediated the association between low self-compassion and depressive symptoms among sexual assault survivors (Hamrick and Owens, 2019). These results indicate that characterological, and to a lesser extent behavioral, self-blame may mediate the link between AISA and negative emotional outcomes. Attesting to the need to test the relative influence of potential mediators, shame mediated the associations between undifferentiated self-blame and posttraumatic stress and depressive symptoms in a sample of sexually assaulted women (Bhuptani and Messman, 2023). Thus, although behavioral and characterological self-blame were not separated, this is preliminary evidence that shame may be a stronger (more proximal) predictor of negative emotional outcomes than characterological and behavioral self-blame.

Informed by cognitive-behavioral models (Ehlers and Clark, 2000; Clark and Beck, 2010), we aimed to ascertain the unique mediating effects of shame, low self-caring, high self-coldness, fear of self-compassion, characterological and behavioral self-blame in the links of AISA with posttraumatic stress, general anxiety, and depressive symptoms by testing these mediators simultaneously. Posttraumatic stress, general anxiety, and depressive symptoms were also tested simultaneously to explore potential differential associations for each, because they may be comorbid but separable responses to AISA (Gong et al., 2019; Strickland et al., 2019). Given studies examining AISA show gender differences or only included women (e.g., Kehayes et al., 2018; Peter-Hagene and Ullman, 2018), gender was controlled. We used a community sample and a cross-sectional mediational design – acceptable when well-founded theories (i.e., the cognitive-behavioral model; Ehlers and Clark, 2000) indicate mediators occur prior to outcomes (Shrout, 2011).

It was hypothesized that:

*H1*: AISA would be significantly positively associated with posttraumatic stress (H1a), general anxiety (H1b), and depressive (H1c) symptoms.

*H2*: AISA would be significantly associated with higher shame (H2a), self-coldness (H2b), fear of self-compassion (H2c), characterological self-blame (H2d), and behavioral self-blame (H2e), and lower self-caring (H2f).

*H3*: In a model with all mediators and outcomes entered simultaneously and controlling for gender, shame (H3a), self-coldness (H3b), low self-caring (H3c), fear of self-compassion (H3d), characterological self-blame (H3e), and behavioral self-blame (H3f) would each partially mediate the association between AISA and all outcomes.

*H4*: Shame would be a stronger mediator than characterological self-blame (H4a) and behavioral self-blame (H4b; Bhuptani and Messman, 2023), self-coldness stronger than low self-caring (H4c; Williamson, 2019), fear of self-compassion stronger than low self-caring (H4d) and self-coldness (H4e; Miron et al., 2016), and characterological stronger than behavioral self-blame (H4f; Peter-Hagene and Ullman, 2018). The strengths of shame compared to self-coldness, low self-caring, and fear of self-compassion, of characterological self-blame compared to fear of self-compassion, self-coldness, and low self-caring, and differences in strength of mediation on each outcome, were not predicted *a priori*, given a lack of previous research.

## Materials and methods

### Participants

Canadian participants (*N* = 409 after excluding 271;^1^ *M* [*SD*] = 28.2 [*4.9*] years old; 51.3% women, 48.7% men; see Table 1 for sample demographics) were obtained through Qualtrics Survey Panels, which recruits from various sources (e.g., website intercept recruitment, member referrals, targeted email lists, gaming sites, customer loyalty web portals, permission-based networks, social media). Qualtrics Survey Panels have been shown to produce valid and reliable data, including in the trauma and addictive behaviors research areas (Walter et al., 2019; Belliveau et al., 2022; Zvolensky et al., 2024). Panel platforms like Qualtrics are also generally viewed to have strengths such as feasibility and efficacy (e.g., Belliveau et al., 2022; Zvolensky et al., 2024). Moreover, some studies have shown data from commercial survey panel pools converge with probability-based and other non-probability derived samples on demographics, mental health, and addictive behavior variables (e.g., Walter et al., 2019; Belliveau et al., 2022). Additionally, Qualtrics Panels implement checks such as attentional/speed checks to help ensure data quality (see footnote 1). Participants were recruited for a gender-balanced and Canada-wide sample. Panelists were eligible if they were 18–35 years old, drank alcohol at least once/month, and had not completed a self-compassion intervention. The minimum alcohol frequency criterion was instituted as non-drinkers could not, by definition, have experienced AISA, and we wanted to ensure we had a sufficiently large number of those with AISA experience given the focus of the study. The specific age span was selected given findings that adult AISA is most common in the 18-35-year-old age range (Status of Women Canada, 2002). As per Qualtrics Survey Panel member agreements, participants were compensated with $6–8 (CAD) in gift cards/other rewards.

**Table 1:** Sample sociodemographic characteristics (*N* = 409).

Characteristic	Frequency (n)	Percentage (%)
Gender (*N* = 409)		
Women	210	51.3%
Men	199	48.7%
Racial background (*N* = 406)		
Caucasian	250	61.6%
Asian	94	23.2%
Middle Eastern/North African	14	3.4%
Hispanic/Latinx/Spanish	9	2.2%
Black/African	9	2.2%
Multiracial	9	2.2%
Indigenous	7	1.7%
Pacific Islander	4	1.0%
Other	10	2.4%
Province of residence (*N* = 409)		
Ontario	206	50.4%
British Columbia	65	15.9%
Alberta	46	11.2%
Quebec	45	11.0%
Atlantic provinces	28	6.8%
Prairie provinces	19	4.6%
Education (*N* = 405)		
University or college degree	224	55.3%
Secondary school or lower	92	22.7%
Graduate degree	51	12.6%
Trade or professional certificate	30	7.4%
Professional degree	8	2.0%
Employment Status (*N* = 407)		
Employed full-or part-time	307	75.5%
Full-or part-time student	43	10.6%
Employed student	10	2.5%
Unemployed	41	10.1%
Other	6	1.5%

### Procedure

Survey panel members received an email invitation or prompt on their respective survey platform informing them of the 30-min survey, specifying the compensation, and providing a survey link. After initial screening, eligible and willing participants completed the survey followed by written debriefing. This study received institutional ethics approval.

### Measures

#### Sociodemographic measure

Sociodemographic questions included age, gender, racial background, province of residence, highest level of education, and employment status.

#### AISA assessment

Frequency of AISA since the age of 18 was measured with the item: “*As a result of using alcohol … I was taken advantage of sexually,”* rated on a 4-point scale from 0 (*never*) to 4 (*more than five times*), part of a larger questionnaire assessing potential drinking-related harms (Strickland et al., 2019). AISA was positively skewed and dichotomized (never [−0.5] vs. once or more [0.5]). The AISA item was positively correlated with general anxiety and depressive symptoms in a previous study, indicative of its validity (Kehayes et al., 2018).

#### Experience of shame scale (ESS)

Shame intensity was assessed with the 25-item ESS (Andrews et al., 2002). Items (e.g., “*Have you felt ashamed of the sort of person you are?*”) were rated on a 4-point scale (1 = *not at all* to 4 = *very much*), referring to the last year. The ESS comprises three types of shame items: characterological (12 items), behavioral (9 items), and body-related shame (4 items). The ESS total correlates with an alternative shame measure (*r* = 0.61; construct validity), shows good test–retest reliability (11 weeks; *r* = 0.83), and excellent internal consistency (Cronbach’ α = 0.92; Andrews et al., 2002; current study α = 0.97).

#### Self-compassion scale (SCS)

Self-compassion was assessed with the 26-item SCS (Neff, 2003). This scale consists of six 4–5 item subcomponents: (1) self-kindness (e.g., “*I try to be loving towards myself when I am feeling emotional pain*”), (2) mindfulness (e.g., “*When something upsets me, I try to keep my emotions in balance*”), (3) common humanity (e.g., “*I try to see my failings as part of the human condition*”), (4) self-judgement (e.g., “*When times are really difficult, I tend to be tough on myself*”), (5) isolation (e.g., “*When I fail at something that’s important to me, I tend to feel alone in my failure*”), and (6) over-identification (e.g., “*When I’m feeling down, I tend to obsess and fixate on everything that’s wrong*”; Neff, 2003). Items were rated on a 5-point scale (1 = *never* to 5 = *almost always*). Using structural equation modelling (SEM), the latent variables of self-caring (scales 1–3 above) and self-coldness (scales 4–6 above) were estimated with their three respective subscales, consistent with evidence supporting a two-factor hierarchical structure of the SCS (Brenner et al., 2017; Strickland et al., 2022). Current study internal consistencies were excellent (self-coldness *α* = 0.93, self-caring *α* = 0.91).

#### Fear of self-compassion scale (FOSCS)

Fear of self-compassion was assessed with the 15-item FOSCS (Gilbert et al., 2011). Items (e.g., *“I feel that I do not deserve to be kind and forgiving to myself”*) were rated using a 5-point scale (0 = *do not agree at all* to 4 = *completely agree*). Factor analyses show the measure is unidimensional (Gilbert et al., 2011). FOSCS scores were associated with self-coldness (*r* = 0.56) and self-caring (*r* = −0.54), supporting construct validity, and the scale has shown excellent internal consistency (i.e., Cronbach’s α = 0.92, Gilbert et al., 2011; current study α = 0.95).

#### Adapted attributional blame questionnaire (ABQ)

Characterological and behavioral self-blame were assessed with a revised version of the ABQ (Tilghman-Osborne et al., 2008). Originally targeting adolescents, our team adapted it for adults by changing the scenarios to ones that would resonate better for emerging/young adults (adapted measure available on request). Following four brief scenarios describing potentially distressing events (e.g., being laughed at for making a mistake during a presentation, under-performing on a group project and receiving a bad evaluation), participants were asked 10 questions with five each addressing characterological self-blame (e.g., “*This happens because I am not a very good worker/person,*”) and behavioral self-blame (e.g., “*I should have worked/tried harder!*”). Questions were rated on a 5-point scale (1 = *definitely would not think* to 5 = *definitely would think*). The original version characterological and behavioral self-blame items loaded on to two distinct factors using exploratory factor analysis (Tilghman-Osborne et al., 2008), and the subscales were positively associated with the State Shame and Guilt Scale (Characterological Self-Blame *r* = 0.47, Behavioral Self-Blame *r* = 0.26), supporting construct validity. The original subscales showed good-to-excellent test–retest reliability (5 months; CSB *r* = 0.98, BSB *r* = 0.94) and internal consistency (Characterological Self-Blame Cronbach’s α = 0.86, Behavioral Self-Blame α = 0.84, Tilghman-Osborne et al., 2008), as did the subscales from the current study’s revised measure (Characterological Self-Blame α = 0.95, Behavioral Self-Blame α = 0.91).

#### Posttraumatic stress disorder checklist – DSM 5 (PCL-5)

Posttraumatic stress symptom severity was assessed with the 20-item PCL-5 (Weathers et al., 2013). Participants were instructed that the items (e.g., “*Repeated, disturbing dreams of the stressful experience*”) represent possible responses to a “very stressful experience,” and to rate how bothersome each symptom was over the past month on a 5-point scale (1 = *not at all* to 5 = *extremely*). Participants completed this measure regardless of AISA status. Factor analyses support a hierarchical structure with six facets (i.e., reexperiencing, avoidance, negative cognitions and mood, anhedonia, dysphoric arousal, and anxious arousal) loading onto a single higher order factor (Blevins et al., 2015); thus, a total score was used in the current study. The PCL-5 correlates with the Posttraumatic Distress Scale (*r* = 0.85), supporting construct validity, and shows good test–retest reliability (1 week; *r* = 0.82) and excellent internal consistency (Cronbach’s *α* = 0.94, Blevins et al., 2015; current study *α* = 0.96).

#### Generalized anxiety disorder−7 (GAD-7)

General anxiety symptom severity was measured with the 7-item GAD-7 (Spitzer et al., 2006). Items (e.g., “*Feeling nervous, anxious, or on edge*”) were rated on a 4-point scale (0 = *not at all* to 3 = *almost every day*) for the past 2 weeks. The scale shows a unidimensional structure (Rutter and Brown, 2017). The GAD-7 was positively correlated with the Beck Anxiety Inventory (*r* = 0.72), supporting construct validity, and showed excellent internal reliability (Cronbach’s α = 0.92, Spitzer et al., 2006; current study α = 0.94).

#### Patient health questionnaire-9 (PHQ-9)

Depressive symptom severity was measured with the 9-item PHQ-9 (Kroenke et al., 2001). Items (e.g., “*Feeling down, depressed, or hopeless*”) were rated on a 4-point scale (0 = *not at all* to 3 = *almost every day*) for the past 2 weeks. The scale shows a unidimensional structure (Keum et al., 2018). The PHQ-9 was positively correlated with the depression subscale of the Short-Form General Health Survey (*r* = 0.73), supporting construct validity, and showed good test–retest reliability (48 h; *r* = 0.84) and good-to-excellent internal consistency (Cronbach’s α = 0.89, Kroenke et al., 2001; current study α = 0.92).

### Analysis plan

Along with correlations (H1 and H2), the mediation model (H3) was tested with the lavaan package in R, using SEM with Maximum Likelihood and full information maximum likelihood estimation for missing data (bootstrapped; 1,000 iterations). The AISA item and gender variables were specified as observed variables. The latent variables characterological self-blame, behavioral self-blame, shame, self-coldness, self-caring, and posttraumatic stress symptom severity were specified using their respective previously supported factors, and fear of self-compassion, severity of general anxiety, and depressive symptoms were specified using three randomly assigned item parcels given their unidimensional structures (see Measures section; Matsunaga, 2008). Mediators were allowed to co-vary. Gender (men and women)^2^ was a covariate for the links between AISA and mediators and outcomes (*a* pathways and direct effects). There were no elevated error covariances among latent variable residuals, indicators of multicollinearity, or problematic skewness (O’Brien, 2007). All reported effects are standardized including the completely standardized indirect effect (ab_cs_). Standardized Root Mean Square Residual (SRMR) ≤ 0.08, Comparative Fit Index (CFI) ≥ 0.90, and Root Mean Square Error of Approximation (RMSEA) ≤ 0.06 were taken as evidence of acceptable to good fit (Hu and Bentler, 1999). The chi-square value was reported (*p* ≥ 0.05 = good fit); however, given its sensitivity to sample size, we used *X*^2^/df < 5.00 (Wheaton et al., 1977; Hu and Bentler, 1999). For H4, the relative strength of each mediator was assessed using percent mediation and Bonferroni corrected *post hoc* difference tests (*p* ≤ 0.0011; 45 tests). A power analysis based on the mediation model showed *N* = 281 was required to detect a small-to-medium effect (0.80 power, *p* < 0.05; Soper, 2021).

## Results

Of the AISA survivors (31.1% of sample), 62% were women and 38% men. Correlations are displayed in Table 2. AISA was significantly positively related to posttraumatic stress, general anxiety, and depressive symptom severity, consistent with H1a-H1c, and to shame, self-coldness, fear of self-compassion, characterological self-blame, and behavioral self-blame, consistent with H2a-H2e (see Table 2). Contrary to H2f, AISA was unrelated to self-caring (see Table 2). All mediators were significantly inter-correlated, except self-caring with fear of self-compassion, characterological self-blame, and behavioral self-blame (see Table 2). The three negative emotional outcomes were significantly inter-correlated (see Table 2). AISA was more common in women, fear of self-compassion higher in men, and self-coldness higher and general anxiety symptoms more severe in women (see Table 2). Surprisingly, posttraumatic stress and depressive symptoms were not more severe in women than men (see Table 2).

**Table 2:** Bivariate correlations between all study variables (*N* = 409).

	M (*SD*)	1	2	3	4	5	6	7	8	9	10
1. AISA	--	--									
2. Shame	58.84 (21.00)	0.40*	--								
3. FOSC	37.74 (14.74)	0.30*	0.64*	--							
4. Self-Coldness	44.27 (10.51)	0.28*	0.66*	0.58*	--						
5. Self-Caring	41.48 (9.37)	−0.03	−0.16*	−0.07	−0.20*	--					
6. CSB	52.43 (18.82)	0.28*	0.50*	0.59*	0.48*	−0.03	--				
7. BSB	62.58 (15.67)	0.28*	0.53*	0.45*	0.49*	0.04	0.73*	--			
8. PTS Sx	48.22 (19.12)	0.44*	0.78*	0.68*	0.59*	−0.01	0.54*	0.50*	--		
9. GA Sx	15.34 (6.30)	0.40*	0.73*	0.46*	0.60*	−0.21*	0.47*	0.48*	0.74*	--	
10. Depressive Sx	18.80 (7.31)	0.37*	0.74*	0.54*	0.58*	−0.17*	0.51*	0.46*	0.75*	0.86*	--
11. Gender	--	−0.15*	−0.01	0.23*	−0.10*	0.09	0.03	−0.06	0.04	−0.16*	−0.04

AISA, alcohol-involved sexual assault, binary coded for those who reported they have (0.5) and have not experienced (−0.5) AISA. Self-coldness is not reverse scored (i.e., higher scores indicate greater self-coldness). FOSC, fear of self-compassion; CSB, characterological self-blame; BSB, behavioral self-blame; PTS, posttraumatic stress; GA, general anxiety; Sx, symptoms. Gender (1 = women, 2 = men). *p < 0.01.

### Mediation model

**Figure 1:**
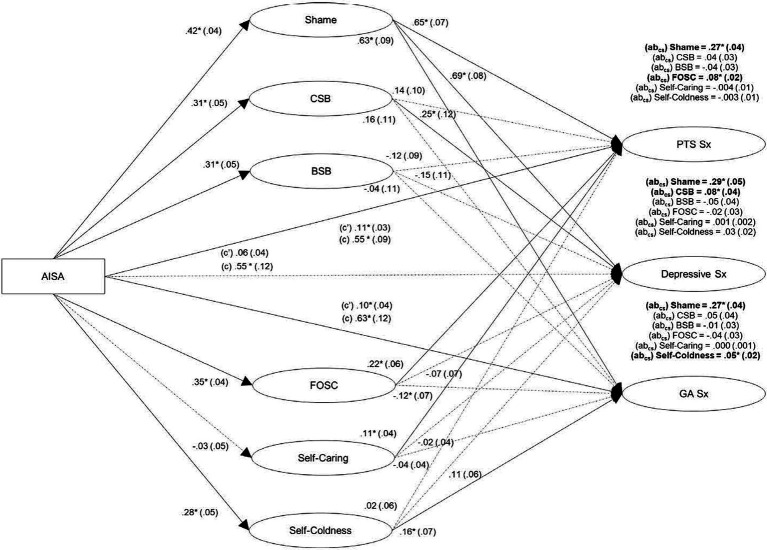
Mediational model with stigma-related mechanisms simultaneously explaining the association between alcohol-involved sexual assault (AISA) and post-traumatic stress (PTS), general anxiety (GA), and depressive symptoms (*N* = 409). Rectangles: observed variables; ovals: latent variables. Gender was controlled but is not shown for simplicity. Effects: ß (*SE*), direct: (c’), total: (c), completely standardized indirect effect: (ab_cs_) above each outcome. AISA, alcohol-involved sexual assault; CSB, characterological self-blame; BSB, behavioral self-blame; FOSC, fear of self-compassion; PTS, posttraumatic stress; GA, general anxiety; Sx, symptoms. Bolded effects, solid lines: significant (*p < 0.05). Model fit statistics: SRMR = 0.06, RMSEA = 0.08, CFI = 0.91, and *X*^2^/df = 3.40 (*X*^2^ [474, *N* = 409] = 1612.75, *p* < 0.001).

Our model with shame, self-coldness, low self-caring, fear of self-compassion, characterological self-blame, and behavioral self-blame simultaneously mediating the associations between AISA and posttraumatic stress, general anxiety, and depressive symptom severity, controlling for gender (see Figure 1) showed acceptable to good fit: SRMR = 0.06, RMSEA = 0.08, CFI = 0.91, and *X*^2^/df = 3.40 (*X*^2^ [474, *N* = 409] = 1612.75, *p* < 0.001). The associations between AISA and negative emotional outcomes were partially (posttraumatic stress and general anxiety symptom severity) to fully (depressive symptom frequency) explained by the set of mediators. Consistent with H3a, shame significantly mediated the associations between AISA and all outcomes, accounting for 42% of the total effect of AISA on general anxiety symptoms, 52% on depressive symptoms, and 49% on posttraumatic stress symptoms. Partially consistent with H3b, self-coldness significantly mediated the associations between AISA and general anxiety symptoms, but not posttraumatic stress or depressive symptoms, accounting for 7% of the total effect of AISA on general anxiety symptoms (vs. only 6% on depressive symptoms and less than 1% on posttraumatic stress symptoms). Contrary to H3c, low self-caring did not significantly mediate any associations, accounting for less than 1% of the total effect of AISA on each outcome. Partially consistent with H3d, fear of self-compassion significantly mediated the associations between AISA and posttraumatic stress symptoms, but not general anxiety or depressive symptoms, accounting for 14% of the total effect of AISA on posttraumatic stress symptoms (vs. only 7% on general anxiety and 4% on depressive symptoms). Partially consistent with H3e, characterological self-blame significantly mediated the association between AISA and depressive symptoms, but not general anxiety or posttraumatic stress symptoms, accounting for 14% of the total effect of AISA on depressive symptoms (vs. only 8% each on general anxiety and posttraumatic stress symptoms). Contrary to H3f, behavioral self-blame did not significantly mediate associations between AISA and any outcome, accounting for only 9% of the total effect of AISA on depressive symptoms, 7% on posttraumatic stress symptoms, and 2% on general anxiety symptoms.

Bonferroni corrected *post hoc* difference tests showed the indirect effect through shame was significantly stronger than through all other mediators for each outcome, including fear of self-compassion (difference = 0.20, *p* < 0.0001) in the case of posttraumatic stress symptom severity, self-coldness (difference = 0.22, *p* < 0.0001) in the case of general anxiety symptom severity, and characterological self-blame (difference = 0.21, *p* < 0.0001) in the case of depressive symptom severity. This pattern is consistent with H4a and H4b. No other indirect effects significantly differed. Thus, while self-coldness mediated the AISA-general anxiety symptom severity link and low self-caring did not, contrary to H4c, the magnitude of mediation between these two mediators did not differ significantly. While fear of self-compassion mediated the AISA-posttraumatic stress symptom severity link and low self-caring did not, contrary to H4d and H4e, fear of self-compassion was not a significantly stronger mediator than either self-caring or self-coldness. While characterological self-blame mediated the AISA-depressive symptom severity link and behavioral self-blame did not, contrary to H4f, the strength of mediation did not differ significantly between them.

## Discussion

This study examined the relative mediating effects of shame, self-coldness, low self-caring, fear of self-compassion, characterological self-blame, and behavioral self-blame on the associations between AISA and posttraumatic stress, general anxiety, and depressive symptom severity, controlling for gender. AISA was positively related to all outcomes and mediators, except self-caring, consistent with H1-H2 (except H2c). The mediation model showed shame and fear of self-compassion partially mediated the association between AISA and posttraumatic symptom severity, shame and self-coldness partially mediated the association between AISA and general anxiety symptom severity, and shame and characterological self-blame fully mediated the association between AISA and depressive symptom severity, partially consistent with H3. These findings fit very well with the recent results of a qualitative study (Strickland et al., 2023) which revealed that AISA survivors’ resilience toward adverse emotional outcomes was undermined by internalized self-blame and low self-compassion. In contrast, survivors’ values (e.g., empathy) fueled their motivation to resist victim-blaming stigma and self-blaming attributions and to instead practice self-compassion, which helped them to counter the negative emotional consequences of AISA.

Supporting cognitive-behavioral models, previous findings, and H3a/H4a-b, shame emerged as the strongest mechanism linking AISA with all outcomes, even after accounting for the other mediators and associations between outcomes (Ehlers and Clark, 2000; Bhuptani and Messman, 2023). Shame may deter disclosing AISA and support seeking, in turn predicting more severe posttraumatic stress, general anxiety, and depressive symptoms (López-Castro et al., 2019). Moreover, the aversive emotional effects of shame may motivate avoidance of reminders of AISA in attempts to reduce future emotional shame experiences, impeding opportunities to reprocess traumatic memories and potentially contributing to posttraumatic stress symptom maintenance (La Bash and Papa, 2014). AISA survivors may also use worry and ruminative processes, both of which characterize general anxiety, to avoid shame, potentially explaining how shame may contribute to more severe AISA-related general anxiety symptoms (Cândea and Szentagotai-Tătar, 2018). Shame may explain the links between AISA and more severe depressive symptoms through shame-related appraisals involving fear of social judgement, which may have negative implications for their sense of self, a central component of depression (Clark and Beck, 2010). Within cognitive-behavioral models of trauma, appraisals precede emotional responses, indicating the emotional component of shame may be most proximal to our outcomes; this is one possible reason why shame was the strongest mechanism in this study, consistent with Bhuptani and Messman (2023). Our results highlight shame may be the most important mechanism to target in reducing AISA-related negative emotional outcomes and increasing survivors’ likelihood to seek subsequent support. In future, a path model could explore a pattern in which trauma occurs first, then negative appraisals are made, which is followed by shame and the specific forms of emotional psychopathology examined here (i.e., general anxiety, depressive, and posttraumatic stress symptoms).

In addition to shame, self-coldness mediated the associations between AISA and general anxiety symptom severity, but not depressive or posttraumatic stress symptom severity, partially supporting H3b. Self-coldness may link AISA and more severe general anxiety symptoms, characterized by worry and fearing future negative experiences, through the self-coldness tendencies of rumination, worry, catastrophizing, and self-criticism (Rutter and Brown, 2017). Indeed, Raes (2010) showed, in separate models, that worry and rumination, central components of general anxiety symptoms, mediated the association between low self-compassion and anxiety symptoms, while only rumination mediated the link between low self-compassion and depressive symptoms. In contrast to Raes (2010) finding and H3b, after accounting for competing mechanisms and the association between outcomes, self-coldness (which has a ruminative aspect) did not significantly mediate the link between AISA and depressive symptom severity in the present study, suggesting other mechanisms may be more important than self-coldness in the AISA-depression link. Additionally, self-coldness did not mediate the link between AISA and posttraumatic stress symptom severity in our study, indicating the AISA-posttraumatic stress symptom severity link may be explained via avoidance-processes (e.g., shame; Ehlers and Clark, 2000; La Bash and Papa, 2014), rather than worry/ruminative self-coldness components. Moreover, self-caring was unrelated to AISA and did not significantly mediate any associations, contrary to H3c. Strickland et al. (2019) showed self-caring counteracted the effects of AISA on anxiety and depression symptoms, suggesting self-caring may function instead as a compensatory resilience factor.

Fear of self-compassion mediated the link between AISA and posttraumatic stress symptom severity, but not general anxiety or depressive symptom severity, partially consistent with H3d. Given their importance in posttraumatic stress disorder, the fear-based (e.g., expecting bad things to happen if self-compassionate) and emotional avoidance processes (e.g., avoiding strong emotions arising from self-compassion; Geller et al., 2019) elements of fear of self-compassion may link AISA particularly to posttraumatic stress symptom severity (Ehlers and Clark, 2000). While shame was a stronger mediator than fear of self-compassion for AISA-related posttraumatic stress symptom severity, shame and fear of self-compassion were highly associated; future longitudinal studies should identify their temporal sequencing. The cognitive elements of fear of self-compassion and shame (e.g., perceiving oneself as undeserving of compassion or social acceptance) may occur simultaneously, though the emotional component of shame may occur later in the mediational chain than fear of self-compassion.

In addition to shame, characterological self-blame fully mediated the association between AISA and depressive symptom severity, but not posttraumatic stress or general anxiety symptom severity, partially consistent with H3e. Contrary to H3f, behavioral self-blame did not significantly mediate any associations. This pattern supports cognitive-behavioral models of trauma and previous studies showing characterological self-blame is more important than behavioral self-blame in predicting negative outcomes following AISA, as characterological self-blame appraisals that relatively fixed character traits caused the trauma may not provide perceived opportunity for change and possible avoidance of future trauma (Janoff-Bulman, 1979; Peter-Hagene and Ullman, 2018). In contrast, characterological self-blame did not mediate the association between AISA and general anxiety symptom severity, suggesting that after accounting for depressive symptom severity, other mechanisms such as the ruminative, worry, and catastrophizing processes involved in self-coldness may explain this link (Raes, 2010). Similarly, Garnefski and Kraaij (2018) found self-blame, rumination, and catastrophizing were associated with both general anxiety and depressive symptoms when not accounting for their comorbidity, though after controlling for their inter-relation, only catastrophizing was related to general anxiety symptoms, and only self-blame was related to depressive symptoms. Finally, characterological self-blame did not significantly mediate the link between AISA and posttraumatic stress symptom severity, contrary to hypotheses and previous findings (Peter-Hagene and Ullman, 2018). In a model with both characterological self-blame and avoidance-based processes, such as fear of self-compassion, the latter may be more important for more severe posttraumatic stress symptoms. This consistent with Ullman et al.’s (2007) findings that when tested simultaneously, avoidance coping, but not characterological or behavioral self-blame, predicted posttraumatic stress symptoms among sexual assault survivors. Together, our results suggest that over and above the competing mediators, characterological self-blame and shame may be key mechanisms to address regarding AISA-related depressive symptom severity.

### Strengths, limitations, and future directions

Current study strengths include examining the *relative* mediating roles of shame, self-coldness, low self-caring, fear of self-compassion, characterological self-blame, and behavioral self-blame on the links between AISA and three specific negative emotional outcomes, controlling for outcome overlap. Though predominantly Caucasian, our community sample of Canadians makes our sample otherwise quite representative thereby enhancing generalizability. Moreover, our relatively large, gender-balanced sample allowed us to: extend to men many prior findings obtained with women-only samples; examine gender differences in the predictor, mediators, and outcomes; and control gender differences in the mediational analyses. Indeed, the inclusion of men makes this study particularly novel.

Nonetheless, several potential limitations suggest avenues for future research. First, our cross-sectional design precludes causal conclusions, and our use of a survey panel may have added error despite our use of quality checks (see Zack et al., 2019; Belliveau and Yakovenko, 2022; Novielli et al., 2023). Second, while there is no gold standard for AISA assessment and while our broad AISA item may have countered the tendency for AISA survivors not to label their experience as sexual assault (Schwarz et al., 2017), our single-item AISA measure also warrants cautious comparison to studies measuring AISA behaviorally and more comprehensively (e.g., Peter-Hagene and Ullman, 2018). Third, we did not assess childhood sexual assault; given the established relations of childhood sexual assault to many of the mediators and outcomes assessed in the present study (e.g., Miron et al., 2016), and links of childhood sexual assault to adult sexual violence experiences (Messman and Long, 2000), future work should replicate the present findings when controlling childhood sexual assault to establish unique links with AISA. Fourth, all participants completed the posttraumatic stress symptom scale regardless of trauma exposure to compare AISA survivor responses to those without this history (similar to Barlow et al., 2017), and the posttraumatic stress symptom reference event, shame, characterological self-blame, and behavioral self-blame measures were not AISA specific, in contrast to other measures like the Trauma-Related Shame Inventory (Øktedalen et al., 2014) and the non-characterological vs. behavioral self-blame differentiated Rape Attribution Questionnaire (Frazier, 2003). Fifth, given no characterological/behavioral self-blame measures feasible for an online survey were available, we modified the Attributional Blame Questionnaire originally developed for adolescents to be useful with adults, potentially introducing error variance; nonetheless, this adapted measure did show good internal consistency and relations with outcomes that were consistent with theory and past work (see above) supporting its validity. Sixth, as we recruited emerging and young adults, the developmental stage where AISA is most likely to occur, results are not necessarily generalizable to those who experienced AISA outside of this age range or who are older now but experienced AISA in their emerging/young adult years. Moreover, while we excluded infrequent drinkers to ensure an adequate number of those who had experienced AISA in our sample, it only takes one drinking episode to qualify for a possible AISA experience. Future studies should determine whether our results extend beyond the young/emerging adult years and to infrequent drinkers and should control for time since assault. Finally, while our sample size was sufficient for structural equation modeling, we may have been underpowered to detect small effects. Future longitudinal studies should explore the potential sequence of the mediators in larger and more diverse samples using a more comprehensive measure of AISA, and AISA-specific measures of posttraumatic stress symptom severity, shame, and characterological and behavioral self-blame. It would be particularly interesting in the future to determine if the model fits equally well for women and men given that women are more likely to report AISA, yet men have been shown to experience stronger emotional sequelae of AISA exposure, possibly due to greater stigma toward sexual assault victimization in men (Kehayes et al., 2018). We did not include non-binary respondents in our analyses given the very small cell size (and thus unreliable data). Thus, it would be useful for future studies to recruit a larger subsample of non-binary individuals, to develop norms on study measures for this understudied group, and to examine our model’s applicability to gender diverse people. Indeed, trans women and men are much more likely to experience both sexual violence generally (Flores et al., 2021) and AISA specifically (Coulter et al., 2015) than their cis-gender counterparts. Other possible models may also be explored (e.g., a moderated mediation model with characterological self-blame mediating the link between AISA and negative outcomes, and the AISA to characterological self-blame link moderated/buffered by self-caring). Finally, the models for general anxiety and posttraumatic stress outcomes showed partial mediation, suggesting additional mechanisms, such as psychological inflexibility or neuroticism and a direct assessment of internalized stigma should be explored in future (Littleton et al., 2009; Boykin et al., 2018).

In conclusion, we examined the relative mediating effects of shame, self-coldness, low self-caring, fear of self-compassion, characterological self-blame, and behavioral self-blame on the associations between AISA and posttraumatic stress, general anxiety, and depressive symptom severity, controlling for gender and outcome inter-associations. Supporting cognitive-behavioral models (e.g., Ehlers and Clark, 2000) and the heightened risk of internalizing AISA-specific stigma (Brown et al., 2018), shame emerged as the strongest and most comprehensive mediator. Along with shame: fear of self-compassion partially explained the association between AISA and posttraumatic stress symptom severity, potentially via avoidance-based processes; self-coldness partially explained the association between AISA and general anxiety symptom severity, potentially via worry-based cognitions; and characterological self-blame fully explained the association between AISA and depressive symptom severity, potentially via negative self-evaluative cognitions. As such, these distinct mechanisms may be important to address in treatment following AISA via evidence-based psychotherapies for trauma (e.g., cognitive processing therapy, prolonged exposure therapy, eye-movement desensitization and reprocessing, cognitive therapy; see Mavranezouli et al., 2020), particularly for those with posttraumatic stress, general anxiety, and/or depressive symptoms or disorders. Specifically, psychotherapy for AISA survivors should emphasize reducing: fear of self-compassion for clients with predominant posttraumatic stress symptoms; self-coldness for clients with predominant general anxiety symptoms; characterological self-blame for clients with predominant depressive symptoms; and shame regardless of predominant type of emotional disorder symptomatology experienced by the client.

## Data availability statement

The raw data supporting the conclusions of this article will be made available by the corresponding author, by reasonable request, and pending institutional ethics board approval.

## Ethics statement

The studies involving humans were approved by a Dalhousie University Research Ethics Board. The studies were conducted in accordance with the local legislation and institutional requirements. The participants provided their written informed consent to participate in this study.

## Author contributions

SS: Conceptualization, Funding acquisition, Investigation, Methodology, Project administration, Resources, Software, Supervision, Writing – original draft, Writing – review & editing. NS: Conceptualization, Data curation, Formal analysis, Investigation, Methodology, Project administration, Visualization, Writing – original draft, Writing – review & editing. RN-A: Conceptualization, Formal analysis, Writing – original draft, Writing – review & editing. CW: Conceptualization, Funding acquisition, Methodology, Writing – original draft, Writing – review & editing.

## Footnotes

^1^During eligibility screening, 73 interested panelists were deemed ineligible for infrequent alcohol use, 29 for being outside the eligible age range, 3 for being from outside Canada, and 58 for the gender quota being fulfilled. An additional 35 were not surveyed as, although they were deemed eligible, they did not consent to the study. From among those surveyed, the following were excluded from analyses: 11 non-binary participants for insufficient *n* for analyses, and 62 for failing a speeder check performed by Qualtrics to ensure the survey was completed in plausible amount of time, thereby improving data quality.

^2^Other genders were excluded due to insufficient *n.*

## References

[ref1] Aakvaag H. F. Thoresen S. Wentzel-Larsen T. Dyb G. Røysamb E. Olff M. (2016). Broken and guilty since it happened: a population study of trauma-related shame and guilt after violence and sexual abuse. J. Affect. Disord. 204, 16–23. doi: 10.1016/j.jad.2016.06.004, PMID: 27318595

[ref2] Andrews B. Qian M. Valentine J. D. (2002). Predicting depressive symptoms with a new measure of shame: the experience of shame scale. Br. J. Clin. Psychol. 41, 29–42. doi: 10.1348/014466502163778, PMID: 11931676

[ref3] Badour C. L. Dutton C. E. Wright J. J. Jones A. C. Feldner M. T. (2020). Shame proneness, negative cognitions, and posttraumatic stress among women with a history of sexual trauma. J. Aggress. Maltreat. Trauma 29, 699–713. doi: 10.1080/10926771.2020.1725211, PMID: 33716493 PMC7954215

[ref4] Barlow M. R. Turow R. E. G. Gerhart J. (2017). Trauma appraisals, emotion regulation difficulties, and self-compassion predict posttraumatic stress symptoms following childhood abuse. Child Abuse Negl. 65, 37–47. doi: 10.1016/j.chiabu.2017.01.006, PMID: 28110110

[ref5] Belliveau J. Soucy K. I. Yakovenko I. (2022). The validity of Qualtrics panel data for research on video gaming and gaming disorder. Exp. Clin. Psychopharmacol. 30, 424–431. doi: 10.1037/pha0000575, PMID: 35511535

[ref6] Belliveau J. Yakovenko I. (2022). Evaluating and improving the quality of survey data from panel and crowd-sourced samples: a practical guide for psychological research. Exp. Clin. Psychopharmacol. 30, 400–408. doi: 10.1037/pha0000564, PMID: 35377694

[ref7] Bhuptani P. H. Messman T. L. (2023). Role of blame and rape-related shame in distress among rape victims. Psychol. Trauma Theory Res. Pract. Policy 15, 557–566. doi: 10.1037/tra0001132, PMID: 34516220

[ref8] Blayney J. A. Read J. P. Colder C. (2016). Role of alcohol in college sexual victimization and post-assault adaptation. Psychol. Trauma 8, 421–430. doi: 10.1037/tra0000100, PMID: 26950014

[ref9] Blevins C. A. Weathers F. W. Davis M. T. Witte T. K. Domino J. L. (2015). The posttraumatic stress disorder checklist for DSM-5 (PCL-5): development and initial psychometric evaluation. J. Trauma. Stress. 28, 489–498. doi: 10.1002/jts.22059, PMID: 26606250

[ref10] Boykin D. M. Himmerich S. J. Pinciotti C. M. Miller L. M. Miron L. R. Orcutt H. K. (2018). Barriers to self-compassion for female survivors of childhood maltreatment: the roles of fear of self-compassion and psychological inflexibility. Child Abuse Negl. 76, 216–224. doi: 10.1016/j.chiabu.2017.11.003, PMID: 29144981

[ref11] Brenner R. P. Vogel D. Credé M. Terence J. G. Kivilghan D. M. (2017). Two is more valid than one: examining the factor structure of the self-compassion scale (SCS). J. Couns. Psychol. 64, 696–707. doi: 10.1037/cou0000211, PMID: 28358523

[ref12] Brown A. L. Horton J. Guillory A. (2018). The impact of victim alcohol consumption and perpetrator use of force on perceptions in an acquaintance rape vignette. Violence Vict. 33, 40–52. doi: 10.1891/0886-6708.33.1.40 29216933

[ref13] Budden A. (2009). The role of shame in posttraumatic stress disorder: a proposal for a socio-emotional model for DSM-V. Soc. Sci. Med. 69, 1032–1039. doi: 10.1016/j.socscimed.2009.07.032 19695754

[ref14] Cândea D. M. Szentagotai-Tătar A. (2018). Shame-proneness, guilt-proneness, and anxiety symptoms: a meta-analysis. J. Anxiety Disord. 58, 78–106. doi: 10.1016/j.janxdis.2018.07.005, PMID: 30075356

[ref15] Carey K. B. Norris A. L. Durney S. E. Shepardson R. L. Carey M. P. (2018). Mental health consequences of sexual assault among first-year college women. J. Am. Coll. Heal. 66, 480–486. doi: 10.1080/07448481.2018.1431915, PMID: 29405862 PMC6311089

[ref16] Clark D. A. Beck A. T. (2010). Cognitive theory and therapy of anxiety and depression: convergence with neurobiological findings. Trends Cogn. Sci. 14, 418–424. doi: 10.1016/j.tics.2010.06.007, PMID: 20655801

[ref17] Coulter R. W. Blosnich J. R. Bukowski L. A. Herrick A. L. Siconolfi D. E. Stall R. D. (2015). Differences in alcohol use and alcohol-related problems between transgender-and non-transgender-identified young adults. Drug Alcohol Depend. 154, 251–259. doi: 10.1016/j.drugalcdep.2015.07.006, PMID: 26210734 PMC4536098

[ref18] Ehlers A. Clark D. M. (2000). A cognitive model of posttraumatic stress disorder. Behav. Res. Ther. 38, 319–345. doi: 10.1016/S0005-7967(99)00123-0 10761279

[ref19] Flores A. R. Meyer I. H. Langton L. Herman J. L. (2021). Gender identity disparities in criminal victimization: National Crime Victimization Survey, 2017-2018. Am. J. Public Health 111, 726–729. doi: 10.2105/AJPH.2020.306099, PMID: 33600251 PMC7958056

[ref20] Frazier P. A. (2003). Perceived control and distress following sexual assault: a longitudinal test of a new model. J. Pers. Soc. Psychol. 84, 1257–1269. doi: 10.1037/0022-3514.84.6.1257, PMID: 12793588

[ref21] Garnefski N. Kraaij V. (2018). Specificity of relations between adolescents’ cognitive emotion regulation strategies and symptoms of depression and anxiety. Cognit. Emot. 32, 1401–1408. doi: 10.1080/02699931.2016.1232698, PMID: 27648495

[ref22] Geller J. Iyar M. M. Kelly A. C. Srikameswaran S. (2019). Barriers to self-compassion in the eating disorders: the factor structure of the fear of self-compassion scale. Eat. Behav. 35:101334. doi: 10.1016/j.eatbeh.2019.101334, PMID: 31491665

[ref23] Gilbert P. (2010). Compassion focused therapy: distinctive features. Oxfordshire, England, UK: Routledge.

[ref24] Gilbert P. McEwan K. Matos M. Rivis A. (2011). Fears of compassion: development of three self-report measures. Psychol. Psychother. Theory Res. Pract. 84, 239–255. doi: 10.1348/147608310X526511, PMID: 22903867

[ref25] Gong A. T. Kamboj S. K. Curran H. V. (2019). Post-traumatic stress disorder in victims of sexual assault with pre-assault substance consumption: a systematic review. Front. Psych. 10:92. doi: 10.3389/fpsyt.2019.00092, PMID: 30918487 PMC6424881

[ref26] Hamrick L. A. Owens G. P. (2019). Exploring the mediating role of self-blame and coping in the relationships between self-compassion and distress in females following the sexual assault. J. Clin. Psychol. 75, 766–779. doi: 10.1002/jclp.22730 30552686

[ref27] Hu L. Bentler P. M. (1999). Cut-off criteria for fit indexes in covariance structure analysis: conventional criteria versus new alternatives. Struct. Eq. Model. 6, 1–55. doi: 10.1080/10705519909540118

[ref28] Janoff-Bulman R. (1979). Characterological versus behavioral self-blame: inquiries into depression and rape. J. Pers. Soc. Psychol. 37, 1798–1809. doi: 10.1037/0022-3514.37.10.1798, PMID: 512837

[ref29] Kehayes I. L. L. Hudson A. Thompson K. Wekerle C. Stuart H. Dobson K.. (2018). The consequences of alcohol-involved sexual victimization in male and female college students. Can. J. Commun. Ment. Health 37, 127–143. doi: 10.7870/cjcmh-2018-014

[ref30] Keum B. T. Miller M. J. Inkelas K. K. (2018). Testing the factor structure and measurement invariance of the PHQ-9 across racially diverse U.S. college students. Psychol. Assess. 30, 1096–1106. doi: 10.1037/pas0000550, PMID: 29565614

[ref31] Kroenke K. Spitzer R. L. Williams J. B. (2001). The PHQ-9: validity of a brief depression severity measure. J. Gen. Intern. Med. 16, 606–613. doi: 10.1046/j.1525-1497.2001.016009606.x, PMID: 11556941 PMC1495268

[ref32] La Bash H. Papa A. (2014). Shame and PTSD symptoms. Psychol. Trauma Theory Res. Pract. Policy 6, 159–166. doi: 10.1037/a0032637

[ref33] Littleton H. Grills-Taquechel A. Axsom D. (2009). Impaired and incapacitated rape victims. Violence Vict. 24, 439–457. doi: 10.1891/0886-6708.24.4.439, PMID: 19694350

[ref34] López-Castro T. Saraiya T. Zumberg-Smith K. Dambreville N. (2019). Association between shame and posttraumatic stress disorder: a meta-analysis. J. Trauma. Stress. 32, 484–495. doi: 10.1002/jts.22411, PMID: 31291483 PMC7500058

[ref35] Mac Ginley M. Breckenridge J. Mowll J. (2019). A scoping review of adult survivors’ experiences of shame following sexual abuse in childhood. Health Soc. Care Community 27, 1135–1146. doi: 10.1111/hsc.12771, PMID: 31157486

[ref36] Matsunaga M. (2008). Item parceling in structural equation modeling: a primer. Commun. Methods Meas. 2, 260–293. doi: 10.1080/19312450802458935

[ref37] Mavranezouli I. Megnin-Viggars O. Daly C. Dias S. Welton N. J. Stockton S.. (2020). Psychological treatments for post-traumatic stress disorder in adults: a network meta-analysis. Psychol. Med. 50, 542–555. doi: 10.1017/S0033291720000070, PMID: 32063234

[ref38] Messman T. Long P. (2000). Child sexual abuse and revictimization in the form of adult sexual abuse, adult physical abuse, and adult psychological maltreatment. J. Interpers. Violence 15, 489–502. doi: 10.1177/088626000015005003

[ref39] Miron L. R. Seligowski A. V. Boykin D. M. Orcutt H. K. (2016). The potential indirect effect of childhood abuse on post-trauma pathology through self-compassion and fear of self-compassion. Mindfulness 7, 596–605. doi: 10.1007/s12671-016-0493-0

[ref40] Neff K. D. (2003). The development and validation of a scale to measure self-compassion. Self Identity 2, 223–250. doi: 10.1080/15298860309027

[ref41] Novielli J. Kane L. Ashbaugh A. R. (2023). Convenience sampling methods in psychology: a comparison between crowdsourced and student samples. Can. J. Behav. Sci. doi: 10.1037/cbs0000394

[ref42] O’Brien R. M. (2007). A caution regarding rules of thumb for variance inflation factors. Qual. Quant. 41, 673–690. doi: 10.1007/s11135-006-9018-6

[ref43] O’Callaghan E. Ullman S. E. (2021). Are all substance-involved sexual assaults alike? A comparison of victim alcohol use, drug use, and combined substance use in sexual assaults. Women Crim. Just. 34, 88–106. doi: 10.1080/08974454.2021.1914284, PMID: 38694969 PMC11060709

[ref44] Øktedalen T. Hagtvet K. A. Hoffart A. Langkaas T. F. Smucker M. (2014). The trauma related shame inventory: measuring trauma-related shame among patients with PTSD. J. Psychopathol. Behav. Assess. 36, 600–615. doi: 10.1007/s10862-014-9422-5

[ref45] Peter-Hagene L. C. Ullman S. E. (2018). Longitudinal effects of sexual assault victims’ drinking and self-blame on posttraumatic stress disorder. J. Interpers. Violence 33, 83–93. doi: 10.1177/0886260516636394, PMID: 26956436 PMC5014733

[ref46] Raes F. (2010). Rumination and worry as mediators of the relationship between self-compassion and depression and anxiety. Personal. Individ. Differ. 48, 757–761. doi: 10.1016/j.paid.2010.01.023

[ref47] Rutter L. A. Brown T. A. (2017). Psychometric properties of the generalized anxiety disorder Scale-7 (GAD-7) in outpatients with anxiety and mood disorders. J. Psychopathol. Behav. Assess. 39, 140–146. doi: 10.1007/s10862-016-9571-9, PMID: 28260835 PMC5333929

[ref48] Schwarz J. Gibson S. Lewis-Arévalo C. (2017). Sexual assault on college campuses: substance use, victim status awareness, and barriers to reporting. Building Healthy Acad. Commun. J. 1, 45–60. doi: 10.18061/bhac.v1i2.5520

[ref49] Shrout P. E. (2011). Commentary: mediation analysis, causal process, and cross-sectional data. Multivar. Behav. Res. 46, 852–860. doi: 10.1080/00273171.2011.606718, PMID: 26736049

[ref50] Soper D. S. (2021). A priori sample size calculator for structural equation models [software]. Available at: https://www.danielsoper.com/statcalc

[ref51] Spitzer R. L. Kroenke K. Williams J. B. Löwe B. (2006). A brief measure for assessing generalized anxiety disorder: the GAD-7. Arch. Intern. Med. 166, 1092–1097. doi: 10.1001/archinte.166.10.1092 16717171

[ref52] Status of Women Canada (2002). Assessing violence against women: A statistical profile. *Government of Canada*. Available at: https://publications.gc.ca/collections/Collection/SW21-101-2002E.pdf

[ref53] Strickland N. J. Nogueira R. MacKinnon S. Wekerle C. Stewart S. H. (2022). Clarifying the factor structure of the self-compassion scale: nested comparisons of six confirmatory factor analysis models. Eur. J. Psychol. Assess. 38, 365–369. doi: 10.1027/1015-5759/a000672

[ref54] Strickland N. J. Tang K. T. Y. Wekerle C. Stewart S. H. (2023). Fostering resilience and countering stigma: a qualitative exploration of risk and protective factors for negative emotional consequences among alcohol-involved sexual assault survivors. Psychol. Trauma Theory Res. Pract. Policy 15, 1012–1021. doi: 10.1037/tra0001300, PMID: 35901425

[ref55] Strickland N. J. Wekerle C. Kehayes I.-L. Thompson K. Dobson K. Stewart S. H. (2019). Self-compassion as a compensatory resilience factor for the negative emotional outcomes of alcohol-involved sexual assault among undergraduates. Int. J. Child Adolesc. Resilience 6, 52–69. doi: 10.7202/1069076ar

[ref56] Tangney J. P. Miller R. S. Flicker L. Barlow D. H. (1996). Are shame, guilt, and embarrassment distinct emotions? J. Pers. Soc. Psychol. 70, 1256–1269. doi: 10.1037/0022-3514.70.6.1256, PMID: 8667166

[ref57] Testa M. VanZile-Tamsen C. Livingston J. Koss M. (2004). Assessing women's experiences of sexual aggression using the sexual experiences survey: evidence for validity and implications for research. Psychol. Women Q. 28, 256–265. doi: 10.1111/j.1471-6402.2004.00143.x

[ref58] Tilghman-Osborne C. Cole D. A. Felton J. W. Ciesla J. A. (2008). Relation of guilt, shame, behavioral and characterological self-blame to depressive symptoms in adolescents over time. J. Soc. Clin. Psychol. 27, 809–842. doi: 10.1521/jscp.2008.27.8.809, PMID: 25419043 PMC4238306

[ref59] Ullman S. E. Townsend S. M. Filipas H. H. Starzynski L. L. (2007). Structural models of the relations of assault severity, social support, avoidance coping, self-blame, and PTSD among sexual assault survivors. Psychol. Women Q. 31, 23–37. doi: 10.1111/j.1471-6402.2007.00328.x

[ref60] Walter S. L. Seibert S. E. Goering D. O’Boyle E. H. (2019). A tale of two sample sources: do results from online panel data and conventional data converge? J. Bus. Psychol. 34, 425–452. doi: 10.1007/s10869-018-9552-y

[ref61] Weathers F. W. Litz B. T. Keane T. M. Palmieri P. A. Marx B. P. Schnurr P. P. (2013). The PTSD checklist for DSM-5 (PCL-5). National Center for PTSD. Available at: www.ptsd.va.gov

[ref62] Wheaton B. Muthen B. Alwin D. F. Summers G. F. (1977). Assessing reliability and stability in panel models. Sociol. Methodol. 8, 84–136. doi: 10.2307/270754

[ref63] Williamson J. R. (2019). Self-compassion differences in those who have experienced sexual assault and non-sexual assault trauma. Gender Women’s Stud. 2:3. doi: 10.31532/GendWomensStud.2.3.003

[ref64] Winders S. J. Murphy O. Looney K. O'Reilly G. (2020). Self-compassion, trauma, and posttraumatic stress disorder: a systematic review. Clin. Psychol. Psychother. 27, 300–329. doi: 10.1002/cpp.2429 31986553

[ref65] Wong N. Kim K. Ren P. Liu W. S. Oh S. S. Strickland N.. (2022). Self-compassion among youth with child maltreatment histories and psychological distress: a scoping review. Int. J. Child Adolescent Resilience 9, 135–166. doi: 10.54488/ijcar.2022.303

[ref66] Zack E. S. Kennedy J. Long J. S. (2019). Can nonprobability samples be used for social science research? A cautionary tale. Survey Res. Methods 13:2. doi: 10.18148/srm/2019.v13i2.7262

[ref67] Zvolensky M. J. Shepherd J. M. Clausen B. K. Robison J. Cano M. Á. de Dios M.. (2024). Posttraumatic stress and probable posttraumatic stress disorder as it relates to smoking behavior and beliefs among trauma exposed Hispanic persons who smoke. J. Behav. Med. 47, 581–594. doi: 10.1007/s10865-024-00480-8, PMID: 38409553

